# The Network Relationship Between the Core Elements of Interpersonal Communication Competence and Depression in Chinese College Students

**DOI:** 10.7759/cureus.79454

**Published:** 2025-02-22

**Authors:** XiaoShuang Li, Ma. Agatha Anne D Guintu

**Affiliations:** 1 Graduate School, Angeles University Foundation, Angeles, PHL; 2 Basic Medicine, ShanDong Second Medical University, WeiFang, CHN; 3 Educational Management, Angeles University Foundation, Angeles City, PHL

**Keywords:** college students, depression, depression prevention, interpersonal communication competence, mental health

## Abstract

Depression is a prevalent mental health problem among college students, often stemming from academic stress, social pressure, and self-regulatory needs. This study explored the intricate network relationships between interpersonal communication competence and depressive symptoms to understand better how these variables interact and influence students' mental health. Using a sample of 486 college students from a university in Shandong, China, this study examined the centrality, connectivity, and predictability between 10 dimensions of interpersonal communication competence and dimensions of depressive symptoms using network analysis. Results indicated that stronger interpersonal communication competences, particularly altercentrism, immediacy, and supportiveness, were protective factors for depression, contributing to emotional support and reduced loneliness. Altercentrism is the highest level of mediation between interpersonal competence and depression. The study identified gender differences, with men relying more on immediacy, while women emphasized interaction management and expressiveness. The findings highlight the importance of gender-specific interventions that address gender-specific needs, and that such interventions can improve interpersonal communication competences as a strategy for alleviating depression in college students.

## Introduction

In recent years, there has been a growing concern about the mental health of college students, and depression has become one of the most prevalent and serious mental health challenges facing this population [1]. The transition to college life often brings additional stressors, including academic pressures, social challenges, and the need for self-adjustment, which can lead to feelings of isolation, low self-esteem, and a lack of emotional support, all of which are major risk factors for depression. Therefore, understanding the underlying factors that contribute to the development and maintenance of depressive symptoms is critical to the development of effective interventions that promote students' mental health.

Interpersonal communication is one of these factors that is receiving increasing attention. Interpersonal communication skills, such as self-expression, empathy, and social relaxation, have been shown to play an important role in shaping an individual's social experience and emotional well-being. Effective interpersonal communication enables individuals to express their emotions, understand others, and build supportive social networks [2], all of which can serve as protective factors against mental health problems, including depression. On the other hand, poor interpersonal communication may lead to feelings of isolation, misunderstanding, and emotional distress, which can exacerbate depressive symptoms [3]. Despite the growing recognition of the importance of interpersonal communication for mental health, the specific network relationship between these communication skills and depressive symptoms remains unclear.

The purpose of this study was to understand the complex, multifaceted interactions between various interpersonal communication competence and depressive symptoms by exploring the network relationships between interpersonal communication skills and depressive symptoms among college students. It provides a reference for universities to alleviate depressive symptoms and improve the mental health of college students by increasing the level of core elements of interpersonal communication competence.

## Materials and methods

Research subjects

This study was conducted with students from a university in Shandong Province, China, and was approved by the university's ethics committee (ERC code: 2024YX218). According to Raosoft's calculations (Raosoft Inc., Seattle, WA), the sample size required to achieve a margin of error of 5% and a confidence level of 95% was 376 students. However, taking into account the possibility of non-response or incomplete surveys, a total of 500 questionnaires will be distributed.

A cross-sectional quantitative survey design using stratified random sampling was used in this study. The students will be stratified according to the number of students in each faculty of the university and within each faculty, simple random sampling will be used to select the students. The number of questionnaires distributed to each faculty will reflect the proportion of students in that faculty to the total number of students. The questionnaire will be administered via the college's student management system and all students who receive the questionnaire will receive a text message reminder informing them of their participation in the survey and the opportunity to win a random prize upon completion. Students who agreed to participate would be entered into the questionnaire and those who refused to participate would not be able to proceed to complete the survey. The questionnaire includes an informed consent form to ensure that students are fully aware of the purpose of the study, the use of data, and the guarantee of confidentiality before they begin. In addition, students who had participated in a similar study covering the same key themes within the last six months were not entered to complete the questionnaire.

Research instruments

Interpersonal Competence Scale

The Interpersonal Communication Competence Scale (ICS) developed by Rubin and Martin includes “self-disclosure,” ”empathy,” “Social Relaxation,” “Assertiveness,” “Altercentrism,” “Interaction Management,” “Expressiveness,” “Supportiveness,” “Immediacy,” and “Environmental Control” 10 dimensions [4], each dimension includes three questions, the whole scale has 30 questions in total. Respondents rated each entry on a Likert scale ranging from 1 (strongly disagree) to 5 (strongly agree), with the score for each entry representing the degree of agreement with the entry. The Cronbach's alpha coefficient for this scale in this study was 0.916, and the reliability of the scale was excellent.

Depression Scale

The Center for epidemiological studies depression (CES-D) scale developed by Radloff [5] was used in this study, which has good causal validity [6]. The scale has 20 entries including “Positive thoughts,” “Cognition,” “Behaviour”, “Somatic Symptoms,” and “Interpersonal Relationships,” “Somatic Symptoms,” and “Interpersonal Relationships.” Subjects rated each entry on a 4-point scale based on symptom severity, ranging from 0 (never) to 3 (almost every day) for a total score of 0 to 60. The higher the score, the more severe the depressive symptoms. The Cronbach's alpha coefficient for the scale in this study was 0.939, which gives the scale excellent reliability.

Data Processing

Network analysis was performed using R 4.2.2 software (The R Foundation, Indianapolis, IN). When the network consists of 10 nodes, a sample size of 500 is usually sufficient to observe high sensitivity, specificity, and side-weight correlations [7], and the sample size in this study was sufficient for data analysis. First, the network structure was constructed using the qgraph package in R [8], and the image least absolute value convergence and selection algorithm was used to remove less intense connectivity, reduce the number of network connections, and highlight the important connectivity in the network for a more intuitive demonstration of the direct relationships between nodes [9]. Mediativity, proximity, and strength are calculated by centralityPlot and the centrality of core elements is evaluated and the top three elements are selected as core elements based on the overall ranking of centrality, which is in line with the approach of Mullarkey et al. [10]. In addition, the networktools 1.5.0 package was used to calculate the bridge centrality metric (bridge connectivity strength), which measures the sum of the edge weights of the nodes and the nodes of the other dimensions; the mgm package was utilized to assess the predictability of the network nodes and the associated stability coefficients were calculated with the help of the bootnet package, where CS coefficients of 0.25 and above indicate a high level of network stability [11]. Since the study variables were self-reported data from the respondents, the results of Harman's one-way method test showed that all six characteristic roots were greater than 1, and the first principal component explained 31.84% of the variance, which was less than 40% of the judgmental criteria; and validated factor analysis of all the data using a one-method latent factor pathway test [12], which showed that this one-way model did not have a good fit index (χ^2^/df = 5.057, CFI = 0.723, TLI = 0.710, RMSEA = 0.091, SRMR = 0.117), indicating that there is no significant common method bias problem in this study.

## Results

In this study, questionnaires were distributed to 500 students in 14 university colleges, and 486 valid questionnaires were obtained with a validity rate of 97.2%. The age of the participants ranged from 18 to 23 years. In terms of gender, 41.2% were male and 58.8% were female. In terms of grade level, 27.4% of the participants were in the first year, 23.0% in the second year, 17.7% in the third year, 21.4% in the fourth year and 10.5% in the fifth year.

Network structure of the main elements of interpersonal communication

**Figure 1:**
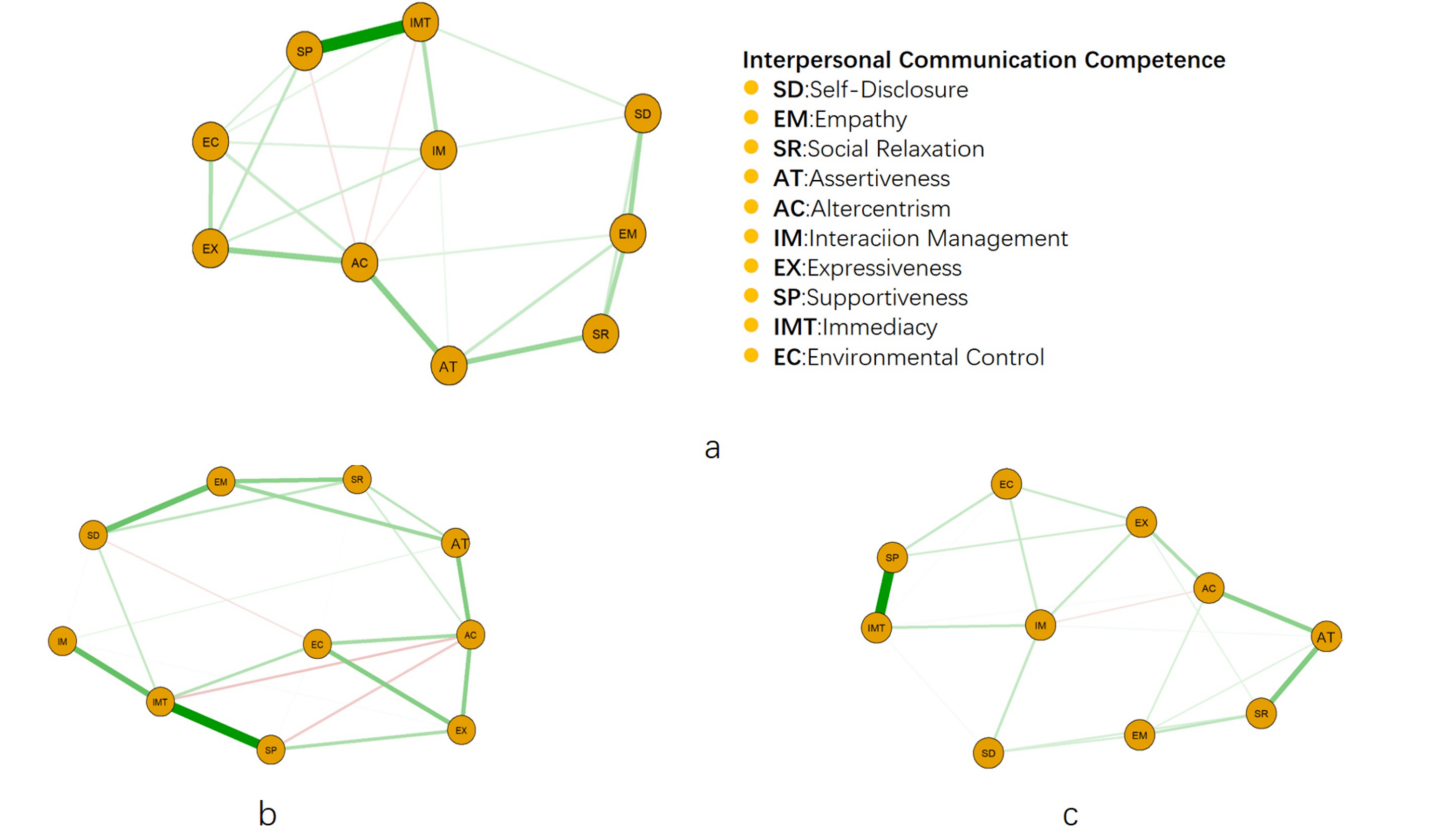
Structure of GLASSO networks for interpersonal interactions among university students. (a) Overall students, (b) male students, and (c) female students. a-c represent the overall, male, and female networks, respectively; the lines between the nodes indicate the bias correlation coefficients of the elements. The thicker the line, the larger the coefficient. The green line indicates a positive correlation, and the red line indicates a negative correlation.

 In the network of the main elements of interpersonal communication, there is a close interaction between some elements, the elements “supportiveness” (SP), “immediacy” (IMT), “altercentrism” (AC), and “assertiveness” (AT) have a strong connection strength and are closely connected (Figure 1a). In the boys' interpersonal network, “supportiveness” (SP), “immediacy” (IMT), “interaction management” (IM), “self-disclosure” (SD), and “empathy” (EM) have strong and tight connections (Figure 1b); and in the girls' interpersonal network, “supportiveness” (SP), “immediacy” (IMT), “assertiveness” (AT), and “social relaxation” (SR) have the strongest connections (Figure 1c). Comparison of the network structures of different genders showed that there was no significant difference in network structure (M=0.153, p>0.05), but there was a significant difference in the overall connection strength (GS male=4.58, GS female=4.30, S=0.29, p<0.05).

Core elements of interpersonal communication for college students

**Figure 2:**
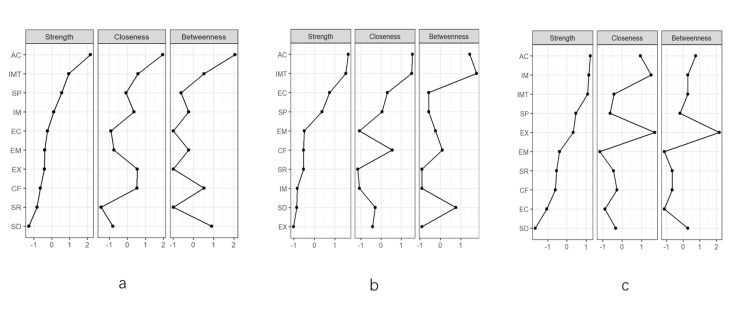
Network centrality of students' interpersonal elements. (a) Overall, (b) male, and (c) female. a-c represent the centrality of overall, male, and female interpersonal elements, respectively. Closeness represents node tightness, Betweenness represents node mediation, and Strength represents node strength.

The core elements of college students' interpersonal communication competence are “altercentrism” (AC), “immediacy” (IMT), and “supportiveness” (SP) (Figure 2a). The core elements of male students are “altercentrism” (AC), “immediacy” (IMT), and “supportiveness” (SP) (Figure 2b). The core elements of female students are “altercentrism” (AC), “interaction management” (IM), and “expressiveness” (EX) (Figure 2c). “Altercentrism” (AC) is the most important factor for all students, but male students focused more on “immediacy” (IMT), while female students focused more on “interaction management” (IM) and “expressiveness” (EX).

Network analysis of interpersonal interaction and depressive symptoms of college students

**Figure 3:**
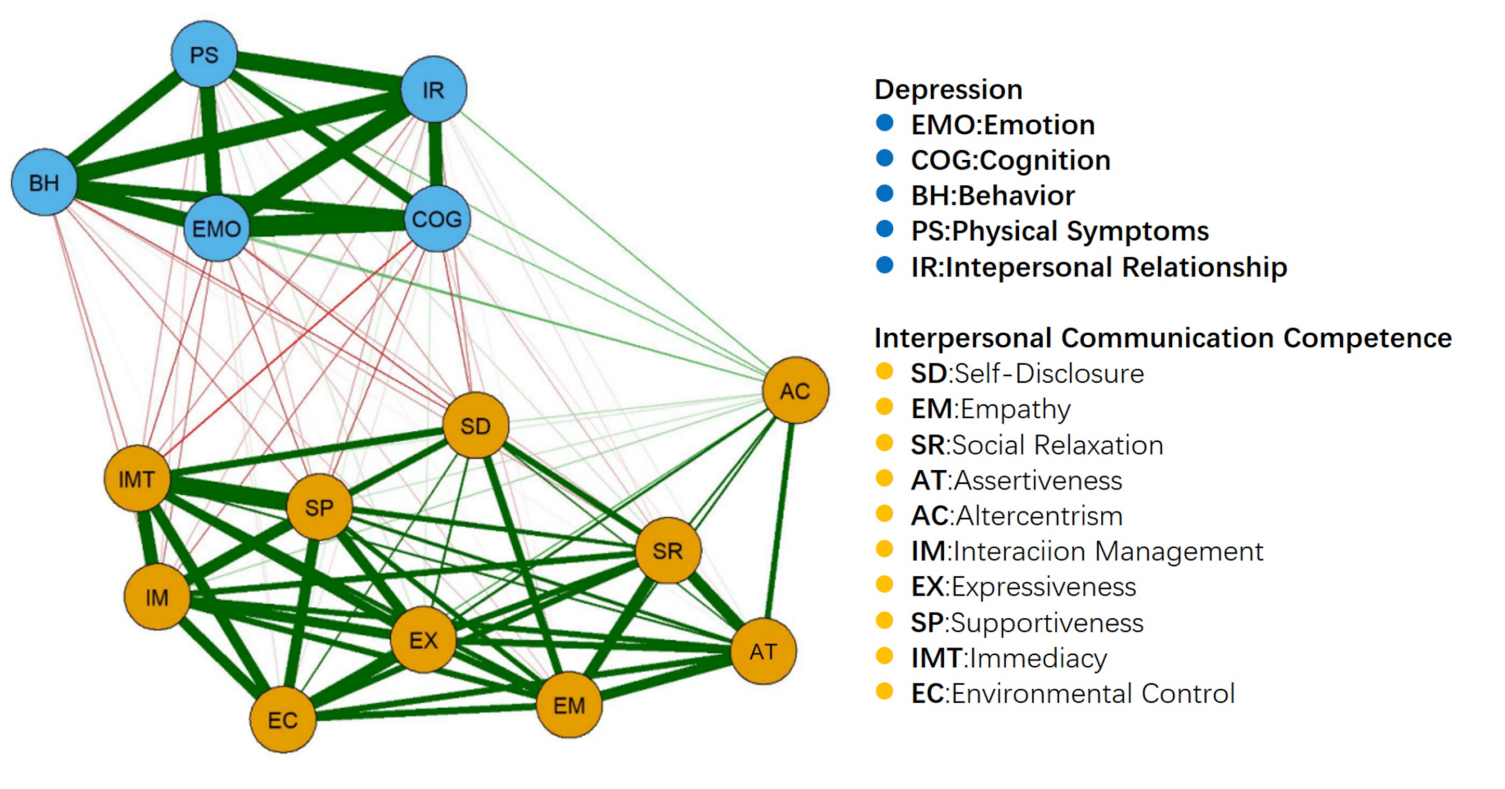
Network model diagram of interpersonal interaction and depressive symptoms. The lines between the nodes indicate the symptom bias correlation coefficients, the thicker the line represents the larger the coefficient, the green line indicates a positive correlation, and the red line indicates a negative correlation.

A Gaussian graphical model was used to construct the undirected network, and to simplify the network, 10 dimensions of interpersonal skills and five dimensions of depression totaling 15 nodes were included in the network. As can be seen in Figure 3, Emotion (EMO) had the strongest link in the network (Str=2.07) and was the most important factor influencing interpersonal competence and depression, and Alternative Centrism (AC) had the highest level of mediation in the network (Bet=45).

Meanwhile, the network structure shows that the six dimensions of interpersonal communication (immediacy, supportiveness, interaction management, environmental control, expressiveness, empathy) are more tightly connected and form a cluster, whereas the five dimensions of depression (emotional, cognition, behavior, physical symptoms, and interpersonal relationship) are more dispersed in their distribution among the nodes, and the strength of connection between the emotional dimension (EMO) and the cognitive dimension (COG) is higher high and tighter. In addition, the predictability of the five nodes of emotion (EMO), cognition (COG), behavior (BH), physical symptoms (PS), and interpersonal relationship (IR) was greater than 0.5, indicating that the depression dimensions were highly predicted by the connected nodes in the network. Among the depression dimensions, the connection strength between the emotion dimension (EMO) and the cognition dimension (COG) was stronger, reflecting the strong correlation between emotional and cognitive factors in depressive symptoms.

**Figure 4:**
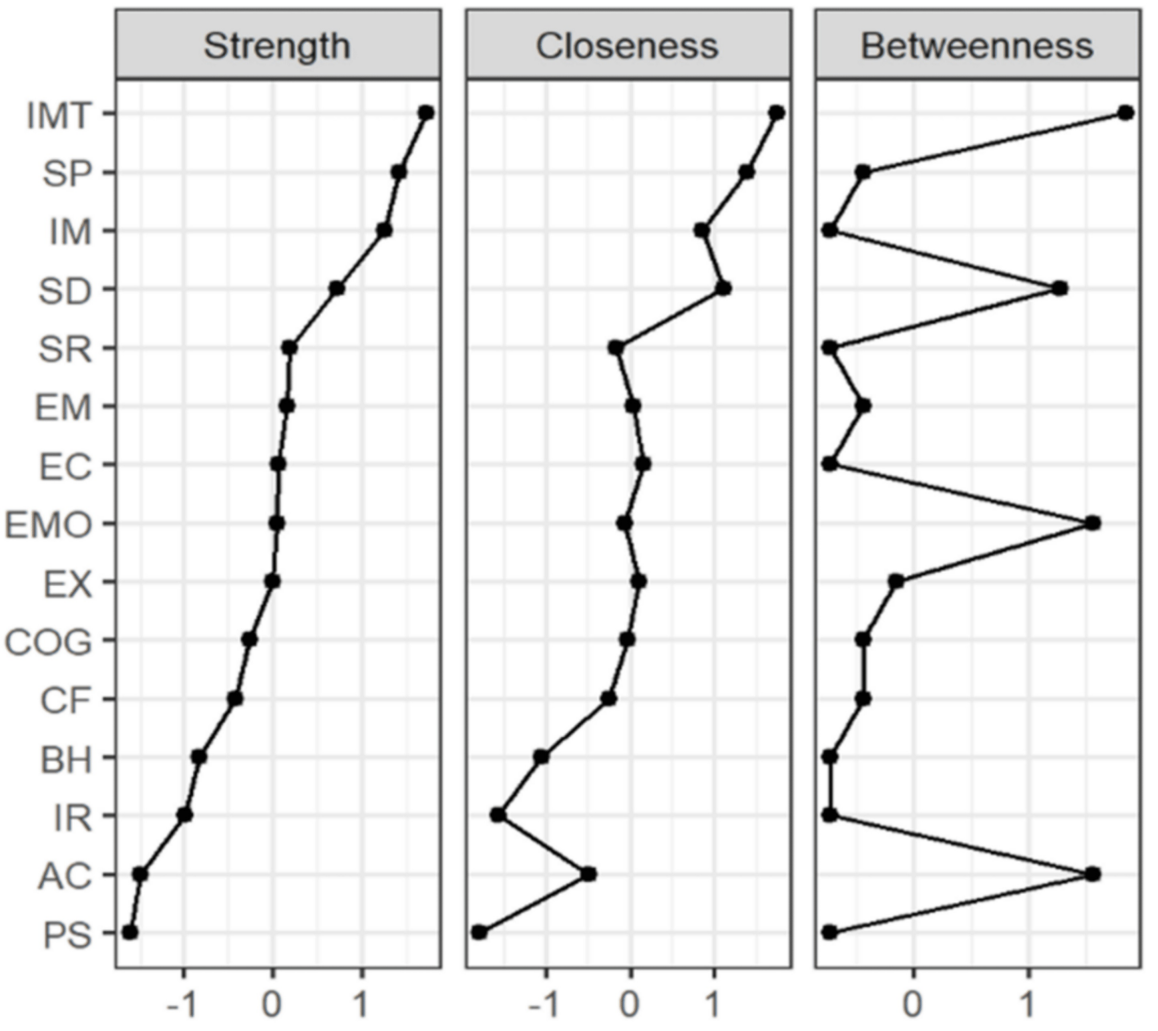
Network centrality indicators of interpersonal interaction and depressive symptoms

As shown in Figure 4, Immediacy (IMT) in the network of interpersonal and depressive symptoms was the center node of this network (Bet=9, Clo=0.0289, Str=6.24).

**Figure 5:**
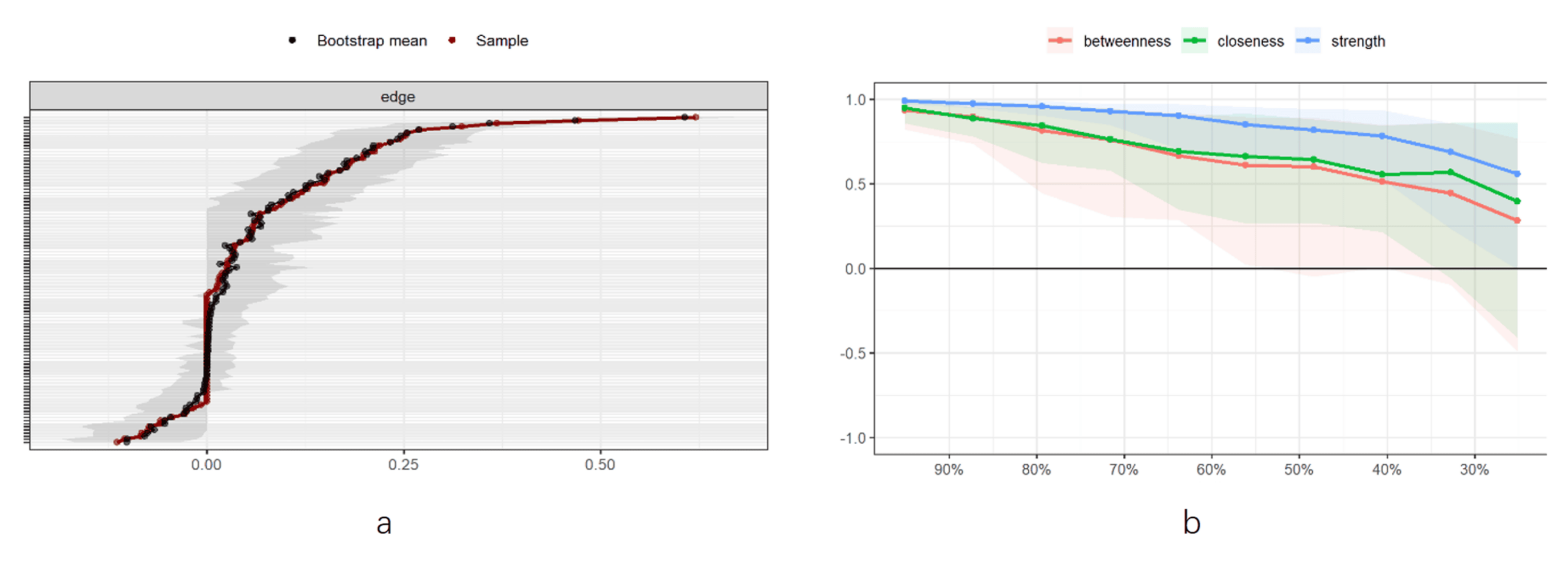
Estimated stability of network structure for interpersonal interactions and depressive symptoms

Meanwhile, the accuracy checks of the network model's edge weights showed that the self-help confidence intervals for most of the edge weights of the above network structure were narrow, indicating that the accuracy of the edge weights in the network was good (Figure 5a). Finally, the results of network stability estimation showed that the node strength had the highest stability, indicating that the network structure of interpersonal and depressive symptoms in this study had high stability (Figure 5b).

The result of the coefficient of the KMO test is 0.957. The value of the coefficient of the KMO test ranges from 0 to 1, and closer to 1 means that the validity of the questionnaire is better. According to the significance of Bartlett's test of sphericity, it can also be seen that the significance of this test is infinitely close to 0, so the questionnaire has good validity (Table 1).

**Table 1:** KMO and Bartlett's test

KMO quantity of sample suitability		0.957
Bartlett's test of sphericity	Approximate chi-square	9,502.580
	Degree of freedom	435
	Significance	0.000

## Discussion

This study explored the relationship between the core elements of college students‘ interpersonal communication competence and depressive symptoms, using network analysis to reveal the intricate dynamic relationship between interpersonal communication competence and depressive symptoms, and by analyzing the interactions and mediating roles between the elements of interpersonal communication competence and the dimensions of depressive symptoms, it provided a reference for understanding the interrelationships between these variables and better understanding the relationship between college students’ interpersonal communication competence and mental health, especially depression.

Interpersonal communication competence as a protective factor against depression

The network structure showed a negative correlation between some elements of interpersonal communication competence (e.g., self-disclosure, empathy, social relaxation, and interaction management) and some depressive symptoms (Figure 3). The negative correlation between these variables suggests that interpersonal communication competence may be a protective factor against depression. This is consistent with previous research that the ability to communicate effectively, understand others' perspectives, and express one's feelings promotes mental health [13]. 

Interpersonal communication provides students with the tools to seek social support, express themselves, and resolve conflict, all of which are critical to mental health. For example, self-disclosure is considered a key factor in fostering close interpersonal relationships, which in turn reduces loneliness [14] - one of the main factors contributing to depression. In addition, empathy and social relaxation can reduce the stressful effects of social situations, which often trigger or exacerbate depressive symptoms [15]. By improving these skills, college students may be able to better cope with the complexity of social relationships, ultimately reducing depression levels.

Altercentrism and depression

Research has found that altercentrism has the highest level of mediation in networks (Bet=45). Altercentrism is the sensitivity of people to other people and the purposes and content of their concerns, people are other-centered, and our information processing is influenced by the pervasive presence of other subjects. Altercentrism is a mediator between interpersonal communication competence with depression may be that people with a strong tendency to alienate are more susceptible to the influence of others, absorbing the pain of others and blurring the boundaries between the self and others, and that this over-conformity with external perspectives may lead to negative self-perceptions, which in turn increase susceptibility to depressive symptoms. Research suggests that a lack of distinction between self and others may impair emotional resilience and make individuals more susceptible to depression when exposed to negative social situations [16]. On the other hand, in positive environments, staying in tune with others may facilitate communication and cooperation, which may alleviate depression. Future research should explore how individual differences in Altercentrism modulate susceptibility to depression, and whether interventions that strengthen the distinction between self and others may mitigate its negative effects.

Depression and its interaction with interpersonal communication competence

The network structure also revealed important links between depressive symptoms and interpersonal communication competence. The strong link between the emotional and cognitive dimensions of depression suggests that emotional and cognitive factors are central to the experience of depression among college students. This is consistent with the existing literature, which suggests that negative mood tends to distort cognitive processes, such as self-perception and social interactions, leading to a cycle of isolation and depressive mood [17]. Furthermore, mediation and proximity analyses suggest that one of the core elements of interpersonal communication competence​​​​​​​, immediacy, plays a key role in the network, both mediating the relationship between interpersonal communication competence​​​​​​​ and depressive symptoms and acting as a protective factor. Immediacy is the ability to engage in meaningful and authentic communication and to connect with others on a deeper level. Students who demonstrate higher immediacy in their interactions may be able to better cope with depressive symptoms by making stronger social connections and fostering emotional support.

The mood dimension of depression was found to be the strongest node in the network, further emphasizing the important role that emotional distress plays in the development and maintenance of depressive symptoms. These findings suggest that interventions aimed at improving interpersonal skills, particularly emotion regulation, and effective communication, may have important therapeutic value for college students suffering from depression.

Gender differences in network structure

An in-depth analysis of the network structure of men's and women's interpersonal competence revealed that men and women did not show significant differences in network structure. However, there is a significant difference (p < 0.05) in the key dimension of overall connection strength. Specifically, there was a significant difference in the degree of connection strength and the degree of activity between nodes in the male and female networks. This significant difference in the dimension of connection strength reveals that men and women differ in the core communication elements of their networks, with male students focusing more on immediacy and female students focusing more on interaction management and expressiveness, and that these differences are consistent with gender expectations of communication styles.

These gender differences suggest that males and females may rely on different interpersonal skills to cope with stress and prevent depression. In social situations, men tend to be more inclined to cope by drawing on certain skills that demonstrate confidence. For example, they may use “alternative-centered” thinking to be open to different perspectives, as well as immediate reactivity to respond quickly to situations, and to appear more confident in social situations. Women tend to exhibit more emotionally expressive behaviors and are more attuned to the emotional cues of others [18]. These findings suggest that different interventions are needed to target male and female students in enhancing interpersonal skills to cope with depression.

Role of immediacy in depression

Immediacy, which includes verbal and non-verbal cues that express warmth and togetherness, is a central node in the network between interpersonal communication skills and depressive symptoms. This finding fits with previous research on the important role that non-verbal cues play in alleviating depressive symptoms by increasing the sense of connectedness between individuals and enhancing social support [19]. Immediacy may play a key role in facilitating positive interpersonal interactions, thereby mitigating the negative effects of depression. Future research could explore the specific mechanisms by which immediacy affects depressive symptoms, especially in the context of college students' peer relationships.

Implications for mental health interventions

The results of this study provide implications for mental health interventions targeting college students in particular to reduce the incidence of depression. Given that interpersonal communication competence​​​​​​​ plays a key role in alleviating depression, educational programs and counseling services should focus on improving these competence​​​​​​​s. Training students to improve competencies in altercentrism, immediacy, supportiveness, interaction management, and expressiveness can help them build stronger social networks, thereby reducing the incidence of depression and improving the mental health of college students.

Limitations and future research

While this study provides implications for the relationship between interpersonal communication competence and depressive symptoms, several limitations need to be addressed in future research. First, the cross-sectional design of this study limits the ability to draw conclusions about the causal relationship between interpersonal communication competence and depression, and longitudinal studies are needed to observe how improving college students' interpersonal competence affects depression. Second, this study was conducted at a university in China and did not involve research studies with college students from different regions, cultural backgrounds, or religions, future research should replicate this study in different regions, cultural environments, or religions to explore whether the network structure identified is universal or context dependent.

## Conclusions

In conclusion, this study identifies the key elements of interpersonal communication competence that affect the mental health of college students through the study of the network relationship between college students‘ interpersonal communication competence and depressive symptoms, and by improving college students’ competence in altercentrism, immediacy, supportiveness, interaction management, and expressiveness, it can help them build stronger social networks, which can reduce the occurrence of depression and improve the mental health of college students. In addition, gender differences should be considered when designing interventions.

## Appendices

Appendix A - interpersonal communication competence scale

INSTRUCTIONS: Here are some statements about how people interact with other people. For each statement, circle the response that best reflects YOUR communication with others. Be honest in your responses and reflect on your communication behavior very carefully,

If you ALMOST ALWAYS interact in this way, circle the 5.

If you communicate this way OFTEN, circle the 4.

If you behave in this way SOMETIMES, circle the 3.

If you act this way only SELDOM, circle the 2.

If you ALMOST NEVER behave in this way, circle 1.

SELF-DISCLOSURE (alpha= 0.63)

1.  I allow friends to see who I really am.

2.  Other people know what I'm thinking.

3.  I reveal how I feel to others.

EMPATHY (alpha= 0.49)

4.  I can put myself in others' shoes.

5.  I don' know exactly what others are feeling.(R)

6.  Other people think that I understand them.

SOCIAL RELAXATION (alpha= 0.63)

7.  I am comfortable in social situations.

8.  I feel relaxed in small group gatherings.

9.  I feel insecure in groups of strangers, (R)

ASSERTIVENESS (alpha= 0.72)

10.  When I've been wronged, I confront the person who wronged me.

11.  I have trouble standing up for myself. (R)

12.  I stand up for my rights.

ALTERCENTRISM (alpha= 0.49)

13.  My conversations are pretty one-sided (R)

14.  I let others know that I understand what they say.

15.  My mind wanders during conversations.

INTERACTION MANAGEMENT (alpha= 0.41)

16.  My conversations are characterized by smooth shifts from one topic to the next.

17.  I take charge of conversations I'm in by negotiating what topics we talk about.

18.  In conversations with friends, l perceive not only what they say but what they don't say.

EXPRESSIVENESS (alpha= 0.46)

19.  My friends can tell when I'm happy or sad.

20.  It's difficult to find the right words to express myself, (R)

21.  I express myself well verbally.

SUPPORTIVENESS (alpha= 0.43)

22.  My communication is usually descriptive, not evaluative.

23.  I communicate with others as though they're equals.

24.  Others would describe me as warm.

IMMEDIACY (alpha= 0.45)

25.  My friends truly believe that I care about them.

26.  I try to look others in the eye when I speak with them.

27.  I tell people when I feel close to them.

ENVIRONMENTAL CONTROL (alpha= 0.60)

 28.  I accomplish my communication goals.

29.  I can persuade others to my position.

30.  I have trouble convincing others to do what I want them to do. (R)

Note 1.  ltems with asterisks are included in the Short-Form (SF) version. All items should be arranged randomly when administered.

Appendix B - The Depression Scale (CES-D)

During the Past Week

Rarely or none of the time (less than 1 day)

Some or a little of the time (1-2 days)

Occasionally or a moderate amount of time (3-4 days)

Most or all of the time (5-7 days)

1. I was bothered by things that usually don’t bother me.

2. I did not feel like eating; my appetite was poor.

3. I felt that I could not shake off the blues even with help from my family or friends.

4. I felt I was just as good as other people.

5. I had trouble keeping my mind on what I was doing.

6. I felt depressed.

7. I felt that everything I did was an effort.

8. I felt hopeful about the future.

9. I thought my life had been a failure.

10. I felt fearful.

11. My sleep was restless.

12. I was happy.

13. I talked less than usual.

14. I felt lonely.

15. People were unfriendly.

16. I enjoyed life.

17. I had crying spells.

18. I felt sad.

19. I felt that people dislike me.

20. I could not get “going.”
